# SGRT‐based DIBH radiotherapy practice for right‐sided breast cancer combined with RNI: A retrospective study on dosimetry and setup accuracy

**DOI:** 10.1002/acm2.13998

**Published:** 2023-04-22

**Authors:** Jianjun Lai, Zhizeng Luo, Haili Hu, Lu Jiang, Jing Wu, Lan Lei, Li Qu, Zhibing Wu

**Affiliations:** ^1^ Instiute of Intelligent Control and Robotics Hangzhou Dianzi University Hangzhou China; ^2^ Department of Radiation Oncology Zhejiang Hospital Hangzhou China; ^3^ Department of Breast Surgery Affiliated Hangzhou First Hospital Zhejiang University School of Medicine Hangzhou China

**Keywords:** DIBH, radiotherapy, right‐sided breast cancer, SGRT

## Abstract

**Background:**

We retrospectively studied the dosimetry and setup accuracy of deep inspiration breath‐hold (DIBH) radiotherapy in right‐sided breast cancer patients with regional nodal irradiation (RNI) who had completed treatment based on surface‐guided radiotherapy (SGRT) technology by Sentinel/Catalyst system, aiming to clarify the clinical application value and related issues.

**Methods:**

Dosimetric indicators of four organs at risk (OARs), namely the heart, right coronary artery (RCA), right lung, and liver, were compared on the premise that the planning target volume met dose‐volume prescription requirements. Meanwhile, the patients were divided into the edge of the xiphoid process (EXP), sternum middle (SM), and left breast wall (LBW) groups according to different positions of respiratory gating primary points. The CBCT setup error data of the three groups were contrasted for the treatment accuracy study, and the effects of different gating window heights on the right lung volume increases were compared among the three groups.

**Results:**

Compared with free breath (FB), DIBH reduced the maximum dose of heart and RCA by 739.3 ± 571.2 cGy and 509.8 ± 403.8 cGy, respectively (*p* < 0.05). The liver changed the most in terms of the mean dose (916.9 ± 318.9 cGy to 281.2 ± 150.3 cGy, *p* < 0.05). The setup error of the EXP group in the anterior‐posterior (AP) direction was 3.6 ± 4.5 mm, which is the highest among the three groups. The right lung volume increases in the EXP, SM, and LBW groups were 72.3%, 69.9%, and 67.2%, respectively (*p* = 0.08), and the corresponding breath‐holding heights were 13.5 ± 3.7 mm, 10.3 ± 2.4 mm, and 9.6 ± 2.8 mm, respectively (*p* < 0.05).

**Conclusions:**

SGRT‐based DIBH radiotherapy can better protect the four OARs of right‐sided breast cancer patients with RNI. Different respiratory gating primary points have different setup accuracy and breath‐hold height.

## 1 INTRODUCTION

In recent years, breast cancer has become the most common malignant tumor in women worldwide.^1^, ^2^, ^3^ The current mainstream treatment mode for breast cancer is still a comprehensive treatment, including surgery, chemotherapy, radiotherapy, targeted therapy, and gene therapy.^4^, ^5^ Postoperative combined radiotherapy can reduce the local and distant recurrence rate, improve the local control rate, and mend the prognosis for patients.^6^, ^7^ In radiotherapy for breast cancer, organs at risk (OARs) and normal tissues around the target region are typically irradiated to a certain extent. Especially for patients with regional nodal irradiation (RNI), the larger irradiation region inevitably expands the volume of normal tissues exposed to radiation while increasing therapeutic benefit, which in turn increases the risk of toxicity.^7^, ^8^

Deep inspiration breath‐hold (DIBH) can significantly ameliorate the risk of cardiotoxicity in patients with left‐sided breast radiotherapy, reduce the irradiation dose to the heart and its substructures amid left‐sided breast radiotherapy,^9^, ^10^ and provide benefit for the lungs, liver, and stomach to varying degrees.^10^, ^11^ Although DIBH is very popular in radiotherapy for left‐sided breast cancer, it is not widely used in radiotherapy for right‐sided breast cancer. Only a few scholars have performed dosimetric studies through the planning system, and a few case reports have been published. These studies have shown that DIBH can provide different degrees of benefit for different patients exposed to radiotherapy for right‐sided breast cancer. In particular, for patients with RNI, DIBH can significantly reduce the maximum dose of the right coronary artery (RCA) and the volume of the liver exposed to low dose.^12^, ^13^

The use of optical surface imaging technology in respiratory‐controlled radiotherapy can dynamically monitor the body surface position and respiratory movement data for patients in real time, thereby allowing non‐ionizing and high‐precision patient positioning, and is widely popular in DIBH gating radiotherapy for breast cancer.^13^, ^14^ Since May 2021, our department has been using surface‐guided radiotherapy (SGRT)–based DIBH radiotherapy for postoperative right‐sided breast cancer patients with RNI. We conducted a retrospective study on the dosimetry and accuracy (setup repeatability and stability) of SGRT‐based DIBH radiotherapy pursuant to real treatment data, to explore the clinical application value and related issues of this technique in radiotherapy for right‐sided breast cancer with RNI.

## 2 METHODS

**FIGURE 1:**
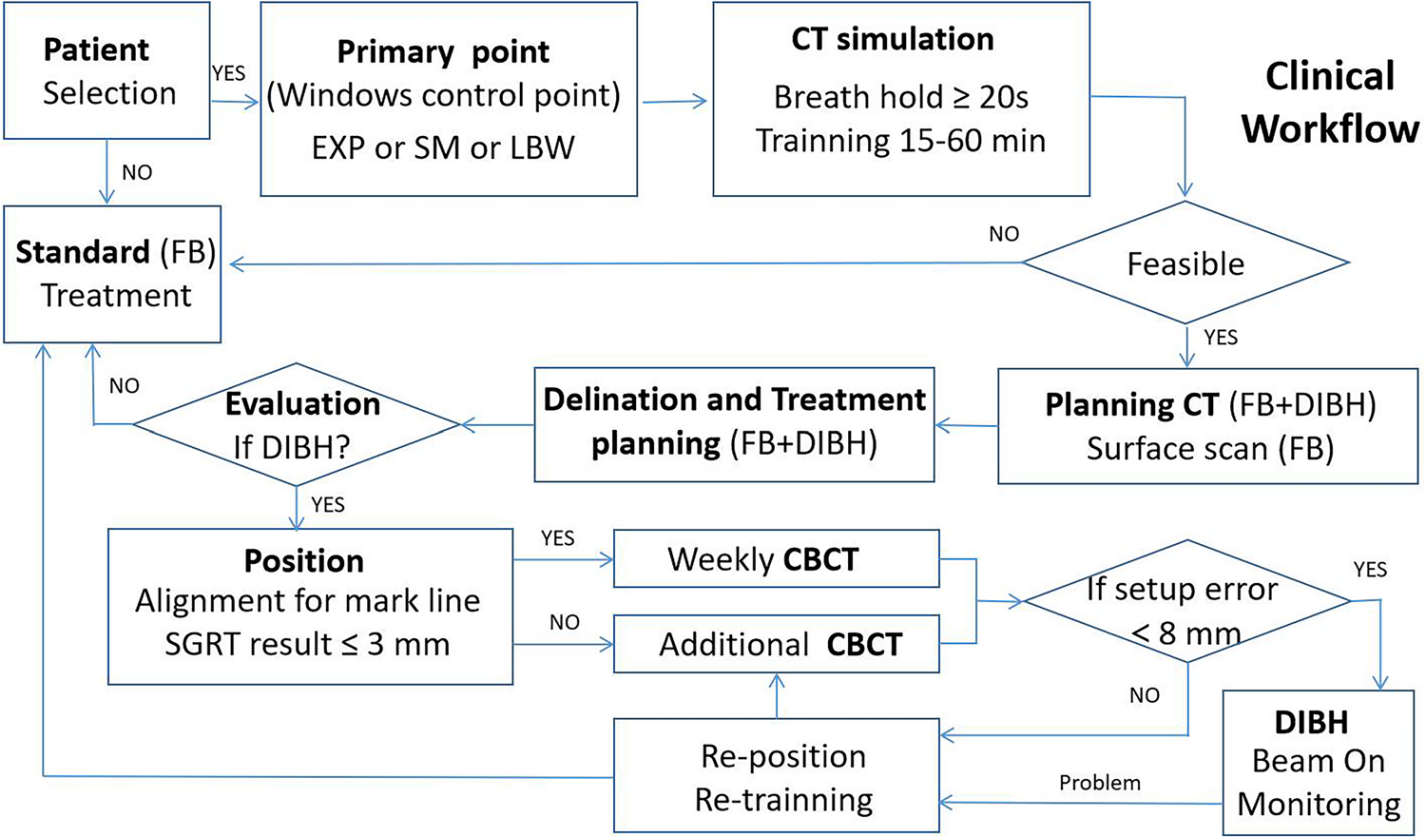
Schematic overview of the clinical workflow. EXP, SM, and LBW represent the gating points placed at the edge of the xiphoid process, sternum middle, and left breast wall, respectively. CBCT, cone beam CT; DIBH, deep inspiration breath‐hold; FB, free breath; and SGRT, surface‐guided radiotherapy.

We included patients who had undergone right‐sided breast radiotherapy (after breast‐conserving or radical surgery) with RNI, who had good compliance and completed the entire DIBH treatment process. A total of 31 patients were screened, comprising 23 patients with breast‐conserving surgery and 8 patients with radical surgery, with an average age of 50.2 years (range, 29−72 years). Figure 1 shows the clinical treatment process of these patients, including respiratory gating primary point setup, respiratory training, CT and optical surface image acquisition, treatment planning, setup and delivery, CBCT position verification, and problem‐handling mechanism.

### 2.1 Immobilization, primary point setup, and CT simulation

**FIGURE 2:**
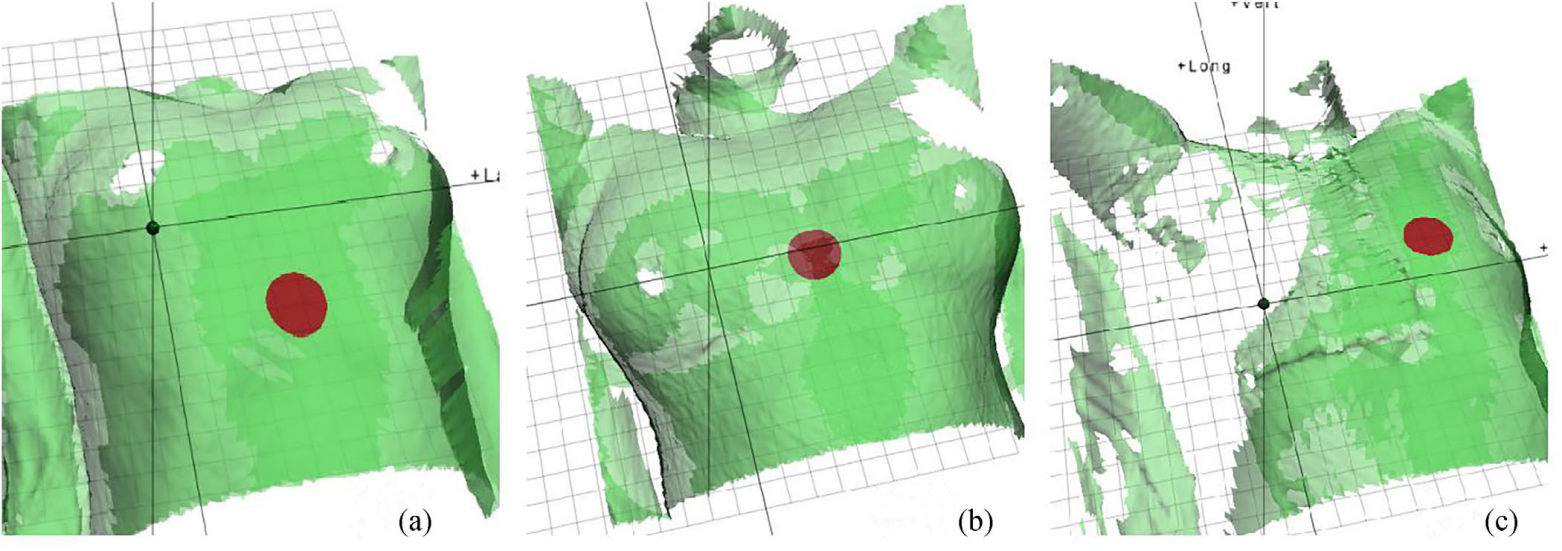
The pictures show the gating primary points for three patients. a, b, and c represent the gating points placed at the edge of the xiphoid process (EXP), the sternum middle (SM), and the left breast wall (LBW), respectively.

For patients who were to receive DIBH radiotherapy for right‐sided breast cancer, individualized negative pressure vacuum bag molds were made for posture fixation and a laser‐based surface scanner (Sentinel, C‐RAD AB, Sweden) was mounted on the CT simulator end for respiratory gating operation. Reference surface were acquired for patients under free breath (FB) on CT end by Sentinel, and the position of gating primary points were set. Changes on the absolute height of the primary point would be used as breath signals to trigger CT acquisition and linear accelerator (LA) beam‐on therapy under breath‐holding. The respiratory gating primary point for breast‐conserving patients was randomly placed at the edge of the xiphoid process or the middle sternum for comparing the setup accuracy under different gating primary points. The gating primary point for radical surgery patients was set at the lateral wall of the left breast to avoid the bolus. Figure 2 shows the locations of the three different gating primary points (EXP, SM, and LBW) for three patients. The patients were trained to perform thoracic breathing and inhale as much as possible; the size of the gating window at the primary point was 1.5−2.5 mm; and it was reckoned that DIBH requirements were met when breath‐holding duration was 20 s and could be repeated for more than three consecutive times. Next, CT images with 3‐mm thickness were acquired under FB and DIBH using a CT simulator (Somatom, Siemens, Erlangen, Germany). Furthermore, reference images of the body surface under FB and the locations of gating primary points were sent to the LA end of an optical‐based surface scanner (Catalyst, C‐Rad AB, Sweden) together with the gating window parameters; and lung volume under FB and DIBH was delineated on CT images using a commercial automatic segmentation tool. According to Oonsiri et al.^15^ and previous DIBH practice experience for left‐sided breast cancer, the target volume of FB and DIBH was delineated and planned simultaneously on the premise that the increases of right lung volume under DIBH is greater than 50% of the right lung volume under FB.

### 2.2 Target volume and OARs delineation, treatment, and planning

For the same patient, the same radiation oncologist delineated the OARs target volume on the CT images under FB and DIBH simultaneously. The gross tumor volume (GTV) and clinical target volume (CTV) were delineated following the Radiation Therapy Oncology Group (RTOG) standard.^16^ PTV and PGTV were defined as the expansion of CTV and GTV by 10 mm and the retraction to 5 mm subcutaneously, respectively. The heart and liver were automatically delineated by an automated segmentation tool and reviewed and manually adjusted by a radiation oncologist, and the RCA was manually delineated.

The prescription dose of PTV was 5000 cGy in 25 fractions in all patients, and PGTV was 5750 cGy divided into 25 fractions for patients after breast conservation. The target volume dose–volume criteria were as recommended by ICRU as follows: maximum dose not exceeding 107%, and coverage of PTV/PGTV by the 95% isodose. FB and DIBH radiotherapy plans were designed by the same radiotherapy physicist using 6 MV‐FFF ray energy for the same patient, and 4−7 tangential intensity modulated radiationtherapy (IMRT) fields based on Dynamic Multi‐Leaf Collimator (DMLC) technology were used depending on the complexity of the plans (as shown in Figure 5). All patients underwent Monte Carlo dose calculation on the radiotherapy planning system (Monaco5.1, Elekta, Stockholm, Sweden). QUANTEC guidelines were used to limit and determine OARs dose–volume, and the dose and volume were kept as low as possible. Both FB and DIBH radiotherapy plans were reviewed and approved by the radiation oncologists. After confirming that the DIBH plan was overall superior to the FB plan, the DIBH radiotherapy plan was sent to the LA end (Infinity, Elekta, Stockholm, Sweden) for plan verification and treatment.

### 2.3 Setup and treatment implementation

For the first treatment, after the laser line in the treatment room was aligned to the body surface marker line of patients under FB, the position was verified using an optical‐based surface scanner (Catalyst, C‐RAD AB, Sweden) at the LA end. The verification criteria were as follows: the deviation of translation ≤3 mm, and the deviation of rotation ≤2°. Optical reference images were subsequently reacquired under FB using Catalyst after setup verification and calibration with CBCT under DIBH. In day‐to‐day treatment, both body surface marker and optical image positioning were performed in accordance with the first treatment. CBCT was used to verify and calibrate the setup under DIBH once a week and to reacquire FB optical reference images. In case of abnormal setup data (≥8 mm) in CBCT, the setup was reverified by CBCT after adjusting position and breathing, the setup was also reverified before the next treatment fraction. The setup error data obtained from automated fusion of CBCT and planning CT by Clipbox registration, and then manually fine‐tuning aligned with the PTV. The Clipbox includes the entire PTV, right lung, sternum, and part of the vertebra. All patients’ treatment with DIBH was triggered by the ResponseTM gating interface within a preset gating window for LA beam‐on therapy. The Catalyst was used to monitor displacement in real time during treatment.

### 2.4 Statistical analysis of dosimetric data and setup accuracy data

Mann–Whitney *U* tests were used to analyze differences in the dose–volume constraints achieved for OARs between the FB and DIBH plans, which was suitable for data that were not normally distributed as confirmed by the Shapiro–Wilk test. Kruskal–Wallis *H* tests were used to analyze differences between multiple groups of sample data, which were also applicable to data that were not normally distributed as confirmed by the Shapiro–Wilk test. The statistical tests were conducted using SPSS27.0, and *p* < 0.05 was considered statistically significant.

## 3 RESULTS

### 3.1 Dosimetric statistics for all DIBH and FB treatment plans

**FIGURE 3:**
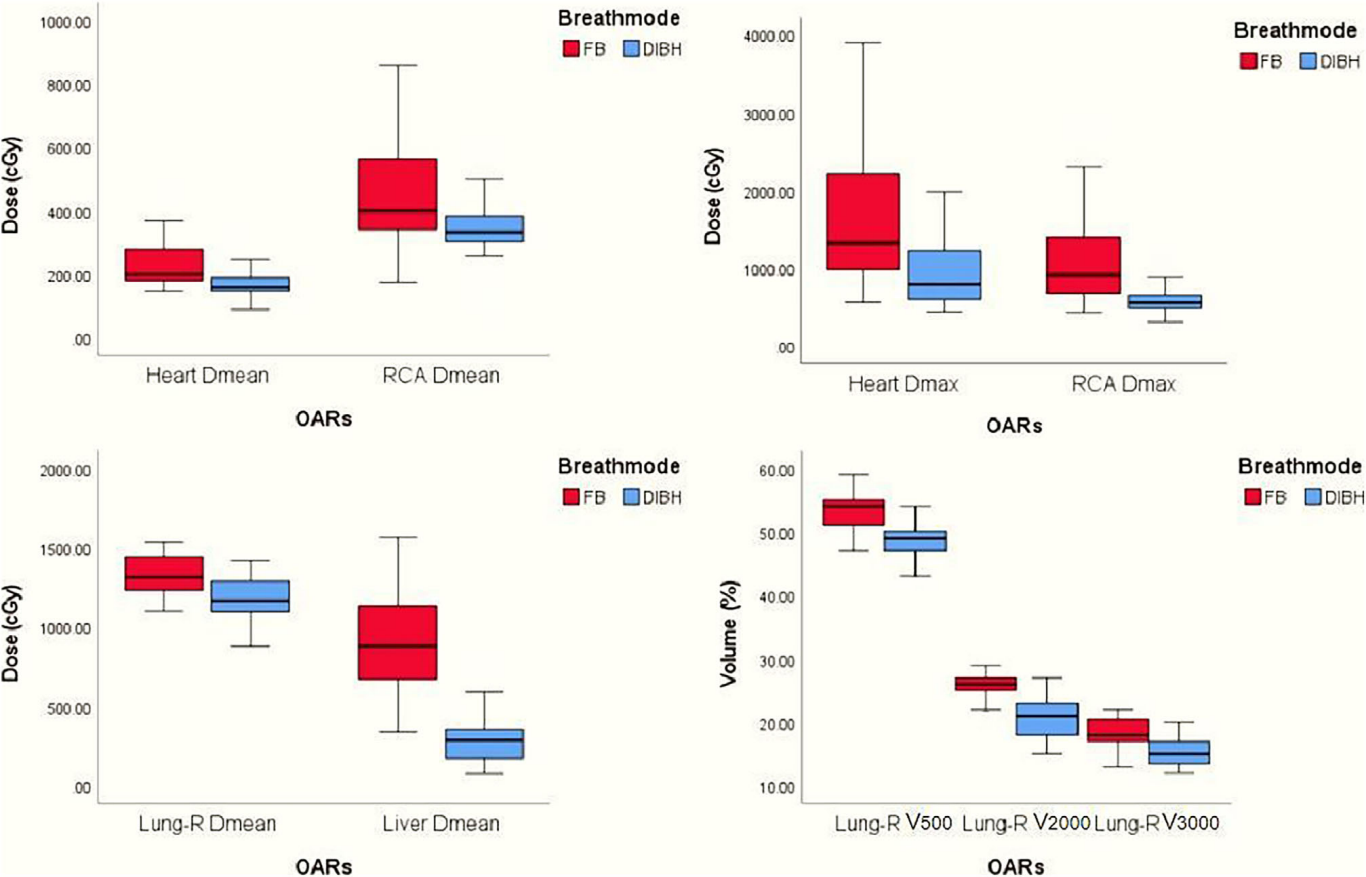
Box plots of the treatment planning data for organs at risk (OARs) for the 31 breast cancer patients included in this study, with deep inspiration breath‐hold (DIBH), free breath (FB), and 4−7 fields tangential IMRT (tIMRT).

In this study, FB and DIBH dosimetric data were collected for 31 patients with right‐sided breast cancer who had completed radiotherapy with DIBH. Coverage of PTV by 95% isodose and coverage of PGTV by 95%−100% isodose were set for both treatment plans. Table 1 summarizes the dose–volume data for four OARs under the premise that the dose–volume requirements of PTV and PGTV were met. Figure 3 shows the box‐plot comparison of the dose–volume data of OARs in 31 patients. The indicators related to target volume dose–volume between the two groups of data met the requirements, and there was no statistically significant difference, while the difference of all OARs dose–volume between the two groups of data was statistically significant.

**TABLE 1:** Summary of treatment planning data for organs at risk for 31 patients with right‐sided breast cancer, with deep inspiration breath‐hold (DIBH), free breath (FB), and 4−7 fields tangential IMRT (tlMRT)

*N* = 31	FB	DIBH	95% CI	*p*	Reduction (%)
Heart mean dose (cGy)	225.2 ± 75.6	171.2 ± 48.9	31.2−96.4	<0.05	24.0
Heart max dose (cGy)	1666.9 ± 961.7	**927.6 ± 390.5**	366.6−1112.1	<0.05	**44.3**
RCA mean dose (cGy)	444.8 ± 161.6	342.4 ± 112.1	31.8−173.2	<0.05	23.1
RCA max dose (cGy)	1090.9 ± 569.5	**581.1 ± 165.7**	296.7−722.6	<0.05	**46.7**
Lung‐R mean dose (cGy)	1332.1 ± 128.7	1165.4 ± 148.6	96.1−237.3	<0.05	12.5
Lung‐R V500 cGy (%)	53.3 ± 3.0	48.3 ± 2.9	3.5−6.5	<0.05	9.4
Lung‐R V2000 cGy (%)	25.7 ± 1.9	20.6 ± 3.2	3.8−6.4	<0.05	19.2
Lung‐R V3000 cGy (%)	18.3 ± 2.5	15.3 ± 2.3	1.7−4.1	<0.05	16.7
Liver mean dose (cGy)	916.9 ± 318.9	**281.2 ± 150.3**	509.1−762.4	<0.05	**69.4**

Dosimetric data are shown as mean values with one standard deviation for OARs. Mann–Whitney *U* tests were used for dosimetric data, and *p* < 0.05 was considered statistically significant. The most favorable values in DIBH were marked in bold. Reduction (%) refers to the average decline for dose–volume data of the four OARs after patients held their breath by a deep inhalation.

#### 3.1.1 Cardiac dose

Compared with FB plan, DIBH reduced the mean dose of the heart and RCA by 54.0 ± 26.7 cGy and 102.4 ± 49.5 cGy, respectively (*p* < 0.05), and the mean reduction rates were 24.0% and 23.1%, respectively. The maximum dose of the heart and RCA decreased by 739.3 ± 571.2 cGy and 509.8 ± 403.8 cGy, respectively (*p* < 0.05), and the average reduction rates were 44.3% and 46.7%, respectively. Only two patients had a decrease in the mean cardiac dose over 100 cGy (131.1 cGy, 100.2 cGy), and three patients had a reduction in their mean RCA dose by more than 200 cGy (249 cGy, 289 cGy, 328 cGy).

#### 3.1.2 Pulmonary dose

According to dose–volume data of the right lung in this study, compared with FB technique, DIBH reduced right lung V500 cGy, V2000 cGy, V3000 cGy, and the mean dose of all patients to varying degrees. V500 cGy decreased from 53.3% ± 3.0% to 48.3% ± 2.9%; V2000 cGy decreased from 25.7% ± 1.9% to 20.6% ± 3.2%; and V3000 cGy declined from 18.3% ± 2.5% to 15.3% ± 2.3%. The mean dose decreased by 166.7 ± 19.9 cGy. In FB mode, there were nine patients whose V3000 cGy exceeded 20%, compared with 31 patients whose V3000 cGy was controlled below 20% by DIBH technique.

#### 3.1.3 Liver dose

The mean liver dose using FB technique was 916.9 ± 318.9 cGy, and the mean liver dose exceeded l000 cGy in four patients. After using DIBH technique, the mean liver dose was only 281.2 ± 150.3 cGy, with an average decrease of 69.4%. Of all 31 patients enrolled in this study, the mean liver dose of three patients decreased by more than l000 cGy (1160 cGy, l005 cGy, 1149 cGy), and the mean liver dose of four patients was controlled below l00 cGy (82 cGy, 91 cGy, 78 cGy, 99 cGy). Hence, DIBH significantly decreased the mean liver dose for patients with right‐sided breast cancer radiotherapy with RNI (*p* < 0.05).

### 3.2 Comparison of setup accuracy under different DIBH gating primary points

The setup accuracy study involved the setup error data calculated from 187 CBCT images over 179 fractions in DIBH mode of 31 patients, including 78 in the EXP group, 65 in the SM group, and 44 in the LBW group. Six patients received 8 ‛additional CBCTs’ over 8 fractions, because of abnormal setup errors. Table 2 shows the absolute values of three‐dimensional setup errors under three different gating primary point placement conditions. When the DIBH gating primary points were placed at the edge of the xiphoid process, sternum middle, and contralateral breast wall, the corresponding overall positioning errors were 3.2 ± 4.0 mm, 2.4 ± 3.0 m, and 2.5 ± 3.2 mm, respectively. The setup accuracy of the gating primary point at EXP was the worst among the three groups. The setup error in the AP direction was 3.6 ± 4.5 mm at EXP, which was the highest among the three groups (3.6 ± 4.5 mm, 2.2 ± 2.6 mm, 2.4 ± 3.1 mm). In the SM group, the setup error interval of 0−3 mm accounted for up to 64.1%, and the abnormal setup error (≥8 mm) only occurred in one fraction (CC direction). In the LBW group, the setup error interval of 0−3 mm made up 59.8%, and the abnormal setup error (≥8 mm) only occurred in one fraction (RL direction). The setup error ranging from 0 to 3 mm accounted for 52.6% in the EXP group, and up to six fractions (2.6%) had abnormal setup error (≥8 mm) for four patients, of which five fractions appeared in the AP direction (Two fractions for one patient, one fractions for each of three patients) and one fraction appeared in the CC directions. DIBH radiotherapy had good setup accuracy overall, but the location of the gating point had a certain impact on the treatment accuracy. The stability and repeatability of the fractional treatment were relatively poor when the gating point was placed at the edge of the xiphoid process.

**TABLE 2:** Summary of setup error data for the 31 right‐sided breast cancer patients included in this study, with deep inspiration breath‐hold (DIBH)

	Directions	EXP (*n* = 11, *f* = 78)	SM (*n* = 12, *f* = 65)	LBW (*n* = 8, *f* = 44)	*p*
Setup error with any directions, mm	All direction	**3.2 ± 4.0**	2.4 ± 3.0	2.5 ± 3.2	<0.05
	AP direction	**3.6 ± 4.5**	2.2 ± 2.6	2.4 ± 3.1	<0.05
	CC direction	3.3 ± 4.0	3.0 ± 3.7	2.8 ± 3.4	<0.05
	RL direction	2.2 ± 2.8	2.3 ± 2.7	2.4 ± 3.0	=0.13
Fractions with any excursion ∈[0, 3) mm, no. (%)	All direction	123 (52.6)	125 (64.1)	79 (59.8)	<0.05
	AP direction	30 (12.8)	48 (24.6)	23 (17.4)	<0.05
	CC direction	32 (13.7)	32 (16.4)	26 (19.7)	<0.05
	RL direction	59 (25.2)	45 (23.1)	30 (22.7)	<0.05
Fractions with any excursion ∈[3, 8) mm, no. (%)	All direction	105 (44.9)	69 (35.4)	52 (38.6)	<0.05
	AP direction	40 (17.1)	17 (8.7)	14 (10.6)	<0.05
	CC direction	45 (19.2)	32 (16.4)	18 (13.6)	<0.05
	RL direction	18 (7.7)	20 (10.3)	19 (14.4)	<0.05
Fractions with any excursion ∈[8,∞) mm, no. (%)	All direction	**6 (2.6)**	1 (0.5)	1 (0.7)	<0.05
	AP direction	**5 (2.2)**	0 (0)	1 (0.7)	<0.05
	CC direction	1 (0.4)	1 (0.5)	0 (0)	<0.05
	RL direction	0 (0)	0 (0)	0 (0)	<0.05

The absolute values of setup error data from CBCT are shown as mean values with one standard deviation. Setup error data were not normally distributed as confirmed by the Shapiro–Wilk test, so Kruskal–Wallis *H* tests were used to analyze differences in setup error data statistics, and *p* < 0.05 was considered statistically significant. The most favorable values in setup error are marked in bold. EXP, SM, and LBW represent the gating points placed at the edge of the xiphoid process, sternum middle, and left breast wall, respectively. AP, CC, and RL represent the anterior–posterior, cranio–caudal, and right–left directions of the patients, respectively.

### 3.3 Lung volume increases and gating primary point height

Table 3 shows the height of gating window and corresponding right lung volume increases. The right lung volume increases of 31 patients included in DIBH treatment process was ≥50%. In the EXP group, the increase averaged 72.3%. The mean height for the gating window from the breathing baseline was 13.5 mm (9.2−20.l mm). In the SM group, the average increase was 69.9%, and the mean height for the gating window from the breathing baseline was 10.3 mm (7.4−16.3 mm). In the LBW group, the average increases was 67.2%, while the mean height for the gating window from the breathing baseline was 9.6 mm (6.5−14.6 mm). The height of the gating window was recorded as the mean value of the upper and lower threshold for the gating window from the breathing baseline. All three groups had good lung volume increases through DIBH, but the height of the gating window was significantly different (*p* < 0.05). The height of the gating window was significantly lower in the SM and LBW groups than in the EXP group.

**TABLE 3:** Summary of the height of gating window and corresponding right lung volume increases for the 31 right breast cancer patients included in this study, with deep inspiration breath‐hold (DIBH)

	EXP (*n* = 11)	SM (*n* = 12)	LBW (*n* = 8)	*p*
Lung‐R volume for FB (cm^3^)	1230.9 ± 184.1	1177.3 ± 186.1	1208.9 ± 178.3	=0.28
Lung‐R volume for DIBH (cm^3^)	2126.1 ± 237.2	1989.0 ± 294.6	2021.6 ± 312.5	=0.36
Lung‐R volume increase rate (%)	72.3	69.9	67.2	=0.08
Gating window height (mm)	13.5 ± 3.7	**10.3 ± 2.4**	**9.6 ± 2.8**	**<0.05**

Lung‐R volume and gating window height data are defined as mean values with one standard deviation. Volume and height data were not normally distributed as confirmed by the Shapiro–Wilk test, so Kruskal–Wallis *H* tests were used to analyze differences in volume and height data statistics, and *p* < 0.05 was considered statistically significant. The most favorable values in gating window height are marked in bold. EXP, SM, and LBW represent the gating points placed at the edge of the xiphoid process, sternum middle, and left breast wall, respectively.

## 4 DISSCUSSION

The toxicity risk of breast cancer radiotherapy is of wide concern among radiation oncologists. Darvby et al.^17^ showed that the incidence of major coronary events after breast cancer radiotherapy linearly increased with the mean cardiac dose. Many studies have shown that amid left‐sided breast cancer radiotherapy, DIBH technology can separate the heart from the mammary gland position, reduce the high dose volume and mean dose of the heart, and reduce the associated risks of heart disease.^18^, ^19^ Meanwhile, coupled with DIBH technology, it can also increase the lung volume and reduce the lung tissue density, which lowers the irradiation dose for the lung,^12^ and reduced the irradiation doses of the contralateral breast, stomach, and liver to varying degrees.^20^, ^21^ Although DIBH technique is widely used for left‐sided breast cancer, there are few reports on the clinical application of DIBH technique in right‐sided breast cancer. According to several radiotherapy planning studies,^13^, ^22^, ^23^, ^24^, ^25^ DIBH reduces lung dose compared with FB in patients with right‐sided breast cancer and achieves liver protection during radiotherapy. A radiotherapy planning study conducted by Pandeli et al.^22^ showed that eight right‐sided breast cancer patients with RNI were treated with DIBH radiotherapy. Their mean dose of the ipsilateral lung decreased from 1820 ± 320 cGy to 1590 ± 230 cGy, and the maximum dose of RCA was reduced from 1160 ± 720 cGy to 560 ± 290 cGy. Although DIBH technology has obvious dosimetric value in right‐sided breast radiotherapy, due to the complexity of the technology and the cost investment of time and labor, not many cases have been reported for clinical application. Abiding by the ‘as low as reasonably achievable’ (ALARA) principle, DIBH radiation therapy for right‐sided breast cancer patients with RNI has been conducted in our department since May 2021, and a retrospective study on dosimetry and setup accuracy for 31 right‐sided breast cancer patients with RNI was conducted.

In this study, the mean cardiac and RCA dose reduction rates by DIBH were 24.0% and 23.1%, respectively. However, DIBH technique in left‐sided breast radiotherapy can reduce the mean dose of the heart and left anterior descending coronary artery (LAD) by 25%−67% and 20%−73%, respectively,^26^ with an average reduction of 46% and 46.5%, respectively. In terms of the contribution of DIBH technology to the average reduction rate for heart and RCA, the benefit for right‐sided breast cancer patients is significantly weaker than that for left‐sided breast cancer patients. As the heart deviates from the right mammary gland, and the mean dose values of the heart and RCA are limited at a low level under FB condition, although the distance between the heart and the chest wall is further increased by DIBH, its contribution to the mean dose reduction of the heart and its substructure is limited. Concerning the maximum dose, DIBH reduced the maximum dose of the heart and RCA, and the average reduction rates were 44.3% and 46.7%, respectively. The results of this study are similar to those of Pandeli et al.^22^ A study by Altinok et al.^27^ showed that high doses to RCA in the heart, especially the proximal part, increased the risk of coronary heart disease. During regional nodal irradiation, internal mammary nodes (IMN) is close to a small amount of heart volume, and DIBH technology can distance IMN from the heart, which contributes to the reduction of the maximum dose to the heart and RCA, and can reduce the risk of coronary heart disease in patients.

The lung is an important OAR in breast cancer radiotherapy. In a meta‐analysis of 742648 breast cancer patients, Grantzau and Overgaard^28^ found that patients who had undergone radiotherapy had a higher secondary lung cancer morbidity 5 and 15 years after treatment (39% and 66%, respectively) than patients who did not receive radiotherapy. Due to advances in technology, the incidence of radiation pneumonitis (RP) after modern breast radiotherapy is lower (1%−5%), and pulmonary function decline is more common, but lung dose is positively associated with the incidence and severity of RP.^29^, ^30^, ^31^ Therefore, breast cancer patients who received extra RNI have a higher rate of RP than those who received whole‐breast radiotherapy alone.^32^, ^33^ In this study, V500 cGy, V2000 cGy, V3000 cGy, and mean dose decreased to different degrees. The results of this study further proved the feasibility and necessity of using DIBH to reduce pulmonary exposure during right‐sided breast radiotherapy with RNI. For the OARs examined in this study, the liver changed the most in terms of the mean dose (916.9 ± 318.9 cGy to 281.2 ± 150.3 cGy) and average dose reduction rate (69.4%). Although radiation‐induced liver toxicity has been widely recognized, related research in breast cancer radiotherapy is still insufficient. Only a few radiotherapy planning studies have shown that DIBH technique can achieve a good liver protection effect in right‐sided breast cancer.^22^, ^23^, ^25^ In the DIBH state, the increase in lung volume makes the liver move away from the target volume, which is the direct reason for the reduction in liver dose. Although the evidence related to the clinical benefit of liver protection in breast cancer radiotherapy is still short,^34^ according to the ALARA principle, the potential benefit of DIBH of right‐sided breast cancer radiotherapy for liver protection is still positive.

With the development of DIBH radiotherapy technology, the repeatability and stability of interfractional and intrafractional breath‐holding have attracted wide concerns.^35^, ^36^, ^37^, ^38^, ^39^ SGRT‐based DIBH radiotherapy adopts optical image‐guided positioning to improve the setup accuracy before treatment, and to monitor patients' position changes in real time during treatment, which enhances the repeatability and stability of the patients' breath‐holding and effectively guarantees treatment accuracy for the patients.^38^, ^39^, ^40^ Amid the implementation of SGRT‐based DIBH radiotherapy, the position of the respiratory gating primary point can be manually placed. In most of the reported studies, the position of the gating primary point is not clear, and there is a lack of relevant comparative studies to guide the setup of the gating primary point.^13^, ^14^, ^40^, ^41^, ^42^ The AAPM Task Group Report 302 believes that a better respiratory curve can be obtained when the gating point is placed at the xiphoid process.^40^ Schönecker et al.^42^ reported that DIBH radiotherapy for left‐sided breast cancer has been performed by setting gating primary points at the sternum. The research team compared and analyzed the CBCT validation data collected from 179 fractions treatment in 31 patients under DIBH after dividing them into three groups (EXP, SM, and LBW), and performed a retrospective evaluation study on the setup accuracy.

**FIGURE 4:**
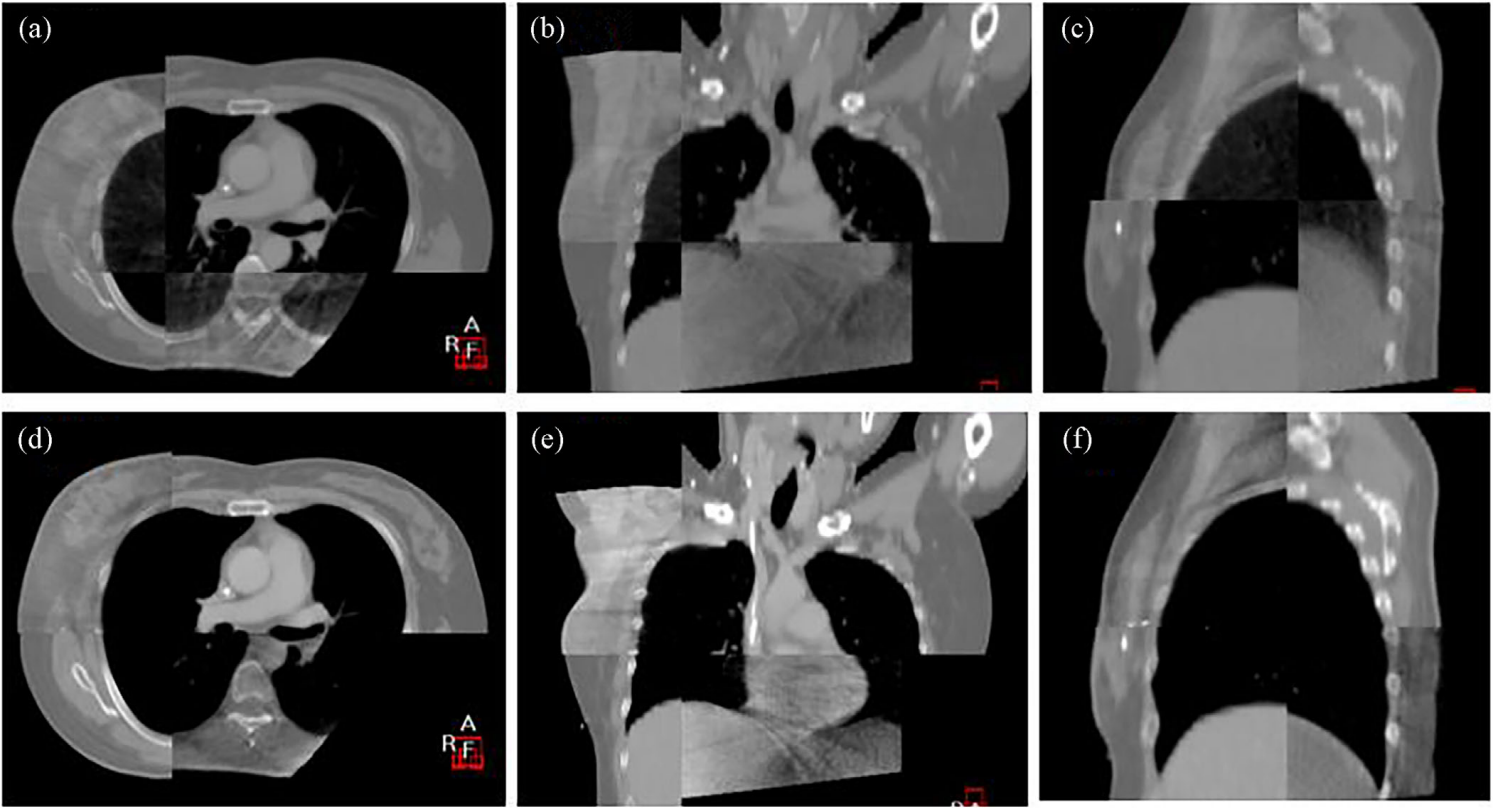
The pictures (a–f) show setup CBCT verification images of a right‐sided breast cancer patient in two different fractions with deep inspiration breath‐hold (DIBH) under the gating points placed at EXP. The transverse plane image (a) shows that insufficient inspiratory volume resulted in excessive setup error in AP direction during treatment, and the sagittal and coronal plane images (b and c) show that insufficient inspiratory volume led to large differences in liver position. However, in another fraction, CBCT images (d–f) show a successful radiotherapy fraction with great DIBH reproducibility.

**FIGURE 5:**
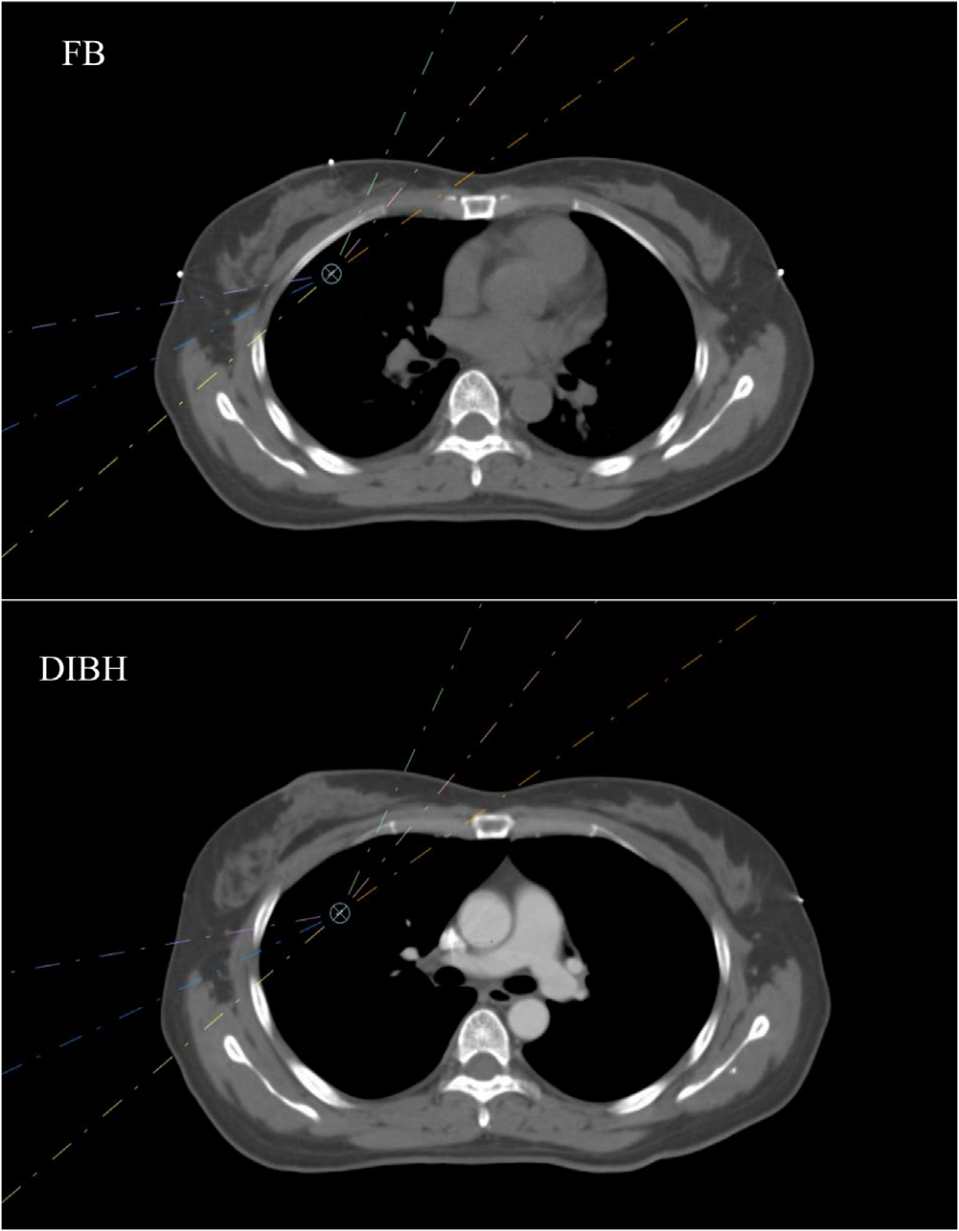
The picture shows the beams arrangement of FB and DIBH radiotherapy plan for the same one patient. FB and DIBH radiotherapy plans were designed by the same radiotherapy physicist using 6 MV‐FFF ray energy, and six tangential intensity modulated radiationtherapy (IMRT) fields based on Dynamic Multi‐Leaf Collimator (DMLC) technology were used for the patient. DIBH, deep inspiration breath‐hold; FB, free breath.

The statistical results of the three sets of data in Table 2 indicated that the SM and LBW groups had better breath‐hold repeatability and stability, and thus higher setup accuracy. In contrast, in the EXP group, six fractions showed abnormal setup error greater than 8 mm, five of them clustered in AP direction. Through the analysis of these abnormal data on CBCT images, we found that inspiratory capacity seriously insufficiently occurred in four patients, and the positions of the heart, lung, and liver were inconsistent with those of localization CT images, as shown in Figure 4. This is an important finding. Although all of the patients were trained to perform thoracic breathing, and CBCT scans were performed while they were holding their breath to a preset range of gating windows, the abnormal breath‐hold stability and repeatability persisted. After analysis, we believes that the height of the gating point in respiration at the edge of the xiphoid process is affected by both the amplitude of thoracic and abdominal respiration. Furthermore, during treatment, patients involuntarily adopt abdominal or mixed breathing to make the breathing amplitude reach the preset range of the gating window, which has led to the relatively poor setup accuracy of the EXP group in this study. For patients with thoracic breathing, the gating point placed at the sternum or breast wall has better interfraction breath‐holding repeatability and stability because the points are mainly affected by the thoracic breathing amplitude and rarely disturbed by abdominal breathing. The analysis of regional anatomical location accuracy was not conducted in this study, so it should be addressed in subsequent studies. Theoretically, the location selection of gating points is equally important to ensure the setup accuracy of DIBH radiotherapy for left‐sided breast cancer. If conditions permit, the abdominal pressure technique and secondary gating point can be used in thoracic breathing to control the occurrence of abdominal breathing for DIBH radiotherapy. We will continue to improve the workflow in DIBH radiotherapy and take careful of use the primary points which place at the EXP (Figure 5).

In addition, we observed obvious differences in DIBH height when different gating points were placed. In traditional DIBH breathing training, the rise height of the gating point after breathing is generally considered an important indicator for predicting lung volume increase. Schröde et al.^43^ did not specify the position of the gating point during the clinical workflow of DIBH but required that the rise height of the DIBH gating point be greater than 12 mm from the baseline. In this study, in the SM and LBW groups with better breath‐holding repeatability and stability, a total of 11 patients did not reach 12 mm in respiration height, the lowest of which was only 6.5 mm, and the right lung volume increases of the patients was above 50%, which was higher than that in Oonsiri^15^ and other studies. Retrospective planning data in Table 1 also confirmed dosimetric benefits of varying degrees in these patients. We believe that the body surface rise in patients after DIBH varies greatly with different patients and different gating points. If the breath‐hold height is used as a reference index to evaluate lung volume increases and if it emerges as the exclusion criterion for DIBH radiotherapy, it is necessary to further study and clarify the specific reference values corresponding to different gating point locations. Until then, it is still recommended to observe the anatomical position changes and calculate the lung volume increases by scanning FB and DIBH CT images for accurate determination.

The results of previous radiotherapy planning studies suggested that patients receiving right‐sided breast radiotherapy with RNI had obvious benefits in heart and lung dosimetry, while patients receiving right‐sided breast radiotherapy without RNI had less benefit.^21^, ^22^, ^23^, ^24^, ^25^ To this end, DIBH radiotherapy was only applied to right‐sided breast cancer with RNI in this retrospective study. The results of this study confirmed the dose reduction of OARs (especially liver) by optical surface image–supported DIBH radiotherapy and proved that DIBH technique had high setup accuracy in right‐sided breast cancer. In the future, DIBH radiotherapy in right‐sided breast cancer without RNI will be studied, to explore its broader clinical application value.

## 5 CONCLUSIONS

Although DIBH radiotherapy has not widely been reported in right‐sided breast cancer, a few of small sample radiotherapy planning studies have shown its potential benefits, especially in right‐sided breast cancer patients with RNI. In this study based on real treatment data, we performed retrospective analysis, which reckoned that on the premise of impeccable workflow, SGRT‐based DIBH radiotherapy could better protect patient's heart, lungs, and liver for right‐sided breast cancer patients with RNI, and interfractions/intrafractions setup had high repeatability and stability to ensure high precision treatment, which is suggested to be popularized.

## AUTHOR CONTRIBUTIONS

The study was conceptualized and the methodology was set up by Lan Lei, Wu Jing, Jianjun Lai, Haili Hu, and Lu Jiang analyzed the data; all authors were involved in the data interpretation. Jianjun Lai and Zhizeng Luo were the major contributors to the manuscript, which was thoroughly reviewed by Zhizeng Luo, Li Qu, and Zhibing Wu. All authors read and approved the final manuscript.

## CONFLICT OF INTEREST STATEMENT

The authors declare no conflict of interest.

## ETHICS STATEMENT

The study was approved by the medical ethics committee of Zhejiang Hospital (No. 20220093K).
